# The one-minute sit-to-stand-test performance is associated with health-related quality of life in patients with pulmonary hypertension

**DOI:** 10.1371/journal.pone.0301483

**Published:** 2024-05-29

**Authors:** Christina Kronberger, Robin Willixhofer, Roya Anahita Mousavi, Mariusz Tadeusz Grzeda, Brigitte Litschauer, Christoph Krall, Roza Badr Eslam

**Affiliations:** 1 Department for Internal Medicine II, Division of Cardiology, Medical University of Vienna, Vienna, Austria; 2 Galen Research, Manchester, United Kingdom; 3 Department for Clinical Pharmacology, Medical University of Vienna, Vienna, Austria; 4 Center for Medical Data Science, Medical University of Vienna, Vienna, Austria; The University of Mississippi Medical Center, UNITED STATES

## Abstract

**Introduction:**

Patients with pulmonary hypertension (PH) have an impaired functional capacity and poor health-related quality of life (HRQoL). The one-minute sit-to-stand test (1-min STST) can be used for the assessment of functional capacity.

**Aims:**

Our aim was to evaluate the 1-min STST performance and its association with patient-reported HRQoL in patients with PH.

**Methods:**

We prospectively assessed functional capacity in 98 PH patients (mean age 66 ± 15 years, 55% female) using the 1-min STST. Patients had to stand up and sit down from a chair as many times as possible within one minute. Patients’ HRQoL was evaluated with the Cambridge Pulmonary Hypertension Outcome Review (CAMPHOR) questionnaire, which consists of the three subcategories symptoms, activities and quality of life (QoL).

**Results:**

We observed a significant correlation of the 1-min STST performance with all HRQoL subcategories assessed with the CAMPHOR questionnaire: A lower number of 1-min STST repetitions correlated with more symptoms (r_s_ = -.398, p < .001), worse functioning (r_s_ = -.551, p < .001) and a decreased QoL (r_s_ = -.407, p < .001). Furthermore, in the multivariable linear regression analysis, adjusted for age, sex, body mass index (BMI) and mean pulmonary artery pressure (mPAP), lower 1-min STST performance was an independent predictor for worse symptoms (est. β = -0.112, p = .003), activities (est. β = -0.198, p < .001) and QoL (est. β = -0.130, p < .001) assessed with the CAMPHOR questionnaire.

**Conclusion:**

Our results indicate that regardless of age, sex, BMI and mPAP the 1-min STST performance is associated with all CAMPHOR HRQoL subcategories in patients with PH. Therefore, the 1-min STST performance might be a new option to assess functional capacity correlated to HRQoL in patients with PH.

## Introduction

Pulmonary hypertension (PH) is a chronic disease characterized by elevated pulmonary artery pressure, which can lead to progressive right ventricular dysfunction and ultimately right heart failure [1, 2]. PH patients exhibit multiple symptoms including dyspnea and fatigue, which in turn cause limitation in functional capacity and significantly reduce patients’ health-related quality of life (HRQoL) [3–7]. The impact of PH on patients’ functional capacity is well recognized, and therefore, the assessment of functional capacity is essential for the diagnosis, management and risk stratification of patients with PH.

Risk stratification via functional capacity assessment is recommended by the current PH guidelines [8] and guides adjustments of medical therapies, thus probably reducing disease burden. In contemporary healthcare, there is a growing emphasis on addressing all facets of a patient’s well-being, including physical, mental, and social aspects [9, 10]. Recognizing the profound impact of PH on functional capacity, which influences all health dimensions, underscores the necessity for a thorough assessment in disease management and risk stratification.

Until now, the six-minute walking test (6MWT) is one of the most commonly used tests to assess functional capacity in patients with PH and is recommended by current guidelines of the European Society of Cardiology (ESC) and the European Respiratory Society (ERS) for the diagnosis and treatment of PH [8].

HRQoL is usually assessed via structured health related patient reported outcome measures. [11] These measures are based on general well-being across patient groups or specific for certain health conditions. For the evaluation of HRQoL in PH, patients receive a validated PH specific survey, the so-called Cambridge Pulmonary Hypertension Outcome Review (CAMPHOR) for a thorough assessment of disease specific HRQoL [12]. Previous studies have shown significant positive correlations between the distance walked in the 6MWT and HRQoL [13–15].

However, there is growing interest in alternative functional capacity tests, such as the one-minute sit-to-stand test (1-min STST), which is of shorter duration, requires less space and is easier to conduct than the 6MWT. In this test participants are encouraged to stand-up and sit-down on a chair as many times as possible within one minute. The 1-min STST was shown to be a reliable and valid measure of functional capacity in patients with various chronic diseases [16–19]. Furthermore, recent studies indicated the feasibility of both tests in patients with PH [20, 21].

Preventive measures, focusing on early identification of worsening disease states, have the potential to reduce the economic burden associated with frequent hospitalizations and treatment expenses for PH patients [22–24]. An extended approach to secondary prevention, utilizing alternative stratification strategies that combine subjective and objective measures, such as HRQoL and functional capacity assessments like the 1-min STST, may support physicians in the timely identification of disease progression.

In the current landscape of PH, the emphasis on HRQoL and functional capacity, particularly through interventions improving functional capacity [25, 26], is acknowledged for its integral role in enhancing the overall well-being of patients with PH. Despite these advancements, there is still limited data regarding the association of functional capacity assessments, specifically using the 1-min STST, with HRQoL in patients with PH.

Therefore, the aim of this prospective study is to evaluate whether there is an association between the 1-min STST performance and HRQoL in patients with PH.

## Methods

### Study design

In this prospective, cross-sectional study, we analyzed data from patients with PH who presented to our center between 03/2020 and 07/2022. PH was diagnosed by a mPAP > 20 mmHg in right heart catheterization [27, 28] in accordance with the current ESC/ERS guidelines for diagnosis and treatment of PH [8]. Patients with orthopedic, vascular, or neurological disorders, who were not able to perform the 1-min STST, were excluded. Participants with language barriers, who could not complete the HRQoL-related questionnaire were likewise excluded.

All patients underwent functional capacity assessment via the 1-min STST and HRQoL assessment via the PH-specific CAMPHOR questionnaire [12].

This study was approved by the local ethics committee (EK#1123/2020) and complies with the principles outlined in the Declaration of Helsinki. All participants provided written informed consent prior to enrollment.

### 1-min STST

The 1-min STST was performed as described by previous studies [20, 29, 30]. Participants were instructed to sit on a standard chair of 45 cm height with their arms folded across their chest and their feet flat on the floor. They were asked to stand up and sit down from the chair as many times as possible within one minute. The number of repetitions completed in one minute was recorded. At test-end the Borg Dyspnea Scale (BDS) was used to assess severity of symptoms of exertional dyspnea [31].

### CAMPHOR questionnaire

The CAMPHOR questionnaire was developed by Galen Research, Enterprise House in 2006 [12]. It has been validated in patients with PH and shows good reliability and validity [12, 14]. The survey contains 65 questions in total, 25 related to symptoms (concerning the frequency and severity of symptoms), 15 related to activities of daily living (the degree to which activities are limited by breathlessness and patients have to rely on outside help to perform the activities of daily living) and 25 related to general quality of life (QoL) (aspects of social and psychological function affected by the disease). According to the formal evaluation strategy, the number of points obtained on each question is summarized to obtain the score for each subcategory (symptoms, activities and QoL). A higher score indicates worse HRQoL and more functional limitation. Questions in the symptom and QoL subcategory are scored from 0–25 points: “yes/true” counts 1 and “no/not true” counts 0 points. Questions in the activity subcategory have three possible responses (able to do on one‘s own without difficulty/able to do on one‘s own with difficulty/unable to do on one’s own, count 0, 1 or 2 points, respectively), resulting in a score that ranges from 0 to 30 points.

### Statistical data analysis

Continuous variables were described by either arithmetic mean and standard deviation (SD), or median and interquartile range (IQR) depending on their distribution. Categorical variables were expressed as frequencies and percentages. Correlations between the number of 1-min STST repetitions and scores in the different categories of the CAMPHOR questionnaire were evaluated using the Spearman’s rank correlation coefficient (*r*_s_).

For box plots, the cut-offs for the HRQoL classes were defined by equal distribution in 25% percentiles (i.e.: quartiles) to differentiate four different grades of impairment with class IV (76–100%) indicating maximum impairment, class III (51–75%) strong impairment, class II (26–50%) moderate impairment and class I (0–25%) minimal to no impairment in each subcategory (symptoms, activity limitation and overall quality of life). Differences in 1-min STST performances between HRQoL classes were analyzed with the Mann–Whitney U test for two classes and with the Kruskal–Wallis (Bonferroni post hoc) test for three or more classes.

To investigate the association between the 1-min STST and the HRQoL, an univariate linear regression for the 1-min STST was tested on the three HRQoL subcategories using Rasch transformed scores [32]. Further, a multivariable analysis, adjusted for age, sex, body mass index (BMI) and mean pulmonary artery pressure (mPAP), was conducted. All statistical analyses were performed using IBM® Statistical Package for Social Sciences (SPSS)® version 27.0, New York, USA and Statistical Software R 4.2.0 under R Studio 2022. A two-sided p-value of less than .05 was considered statistically significant. No adjustment of p-values for multiple hypothesis testing was performed due to the explorative character of the study.

## Results

### Baseline characteristics

A total of 98 patients with PH, with a mean age of 66 ± 15 years, 54 (55%) females, were enrolled in the study. 49 patients (50%) were classified as World Health Organization functional class (WHO-FC) III characterized by marked limitation of physical activity with no discomfort at rest but less than ordinary physical activity causing undue dyspnea. The median N-terminal prohormone of brain natriuretic peptide (NT-proBNP) level was markedly elevated at 1052 pg/mL (IQR 223–2512), indicating the severity of patients’ condition and high-risk of death according to current PH guidelines [8]. The mean number of 1-min STST repetitions was 17 ± 7. The mean BDS score after the 1-min STST was 5.0 ± 2.3. Concerning the subcategories of the CAMPHOR, median scores were 8.5 (IQR 5–14) for symptoms, 7.0 (IQR 4–15) for limitations in activities of daily living, and 5.0 (IQR 1–10) for QoL. Detailed baseline characteristics are shown in **Table 1**.

**Table 1:** Patient characteristics.

Variable	All patients (n = 98)
**Patient characteristics**	**Mean (SD)**
Age, y	66 (15)
Female sex, n (%)	54 (55)
BMI, kg/m^2^	28 (6.9)
NT-proBNP*, pg/mL	1052 [223–2512]
WHO functional class	
Class I, n (%)	11 (11) 32 (33) 49 (50) 6 (6.1)
Class II, n (%)	
Class III, n (%)	
Class IV, n (%)	
**Cardiovascular risk factors**	
Smoking (ex and current), n (%)	56 (57)
COPD, n (%)	22 (22)
Diabetes mellitus, n (%)	31 (32)
Coronary heart disease, n (%)	25 (26)
Arterial hypertension, n (%)	64 (65)
Hyperlipoproteinemia, n (%)	59 (60)
Atrial fibrillation, n (%)	42 (43)
**Test outcomes**	
1-min STST, repetitions	17 (7)
BDS score at test-end	5.0 (2.3)
**CAMPHOR score**	
Symptoms*	8.5 [5 – 14]
Activities*	7.0 [4 – 15]
QoL*	5.0 [1 – 10]
**Right heart catheterization**	
mPAP, mmHg	41 (14)
PCWP, mmHg	15 (8.2)
LVEDP^†^, mmHg	17 (6.8)
PVR, Wood units	5.7 (4.0)
Cardiac output, L/min	4.9 (1.4)

Continuous variables are presented as mean and standard deviation, categorical data are shown as numbers and percentages.

* NT-proBNP levels as well as CAMPHOR subcategory scores are presented as median [IQR]

^†^ LVEDP was not measured in 12 patients

**Abbreviations.** SD = standard deviation; BMI = body mass index; NT-proBNP = N-terminal prohormone of brain natriuretic peptide; WHO = World Health Organization; COPD = chronic obstructive pulmonary hypertension; 1-min STST = one-minute sit-to-stand test; BDS = Borg Dyspnea Scale; QoL = quality of life; mPAP = mean pulmonary artery pressure; PCWP = pulmonary capillary wedge pressure; LVEDP = left ventricular end-diastolic pressure, PVR = pulmonary vascular resistance; IQR = interquartile range.

### Correlation of the 1-min STST results and HRQoL scores

There was an inverse correlation between the number of achieved 1-min STST repetitions and the subcategories of the CAMPHOR. Patients with lower 1-min STST results showed significantly more symptoms (r_s_ = -.398, p < .001), relied more on outside help to be able to perform the activities of daily living (r_s_ = -.551, p < .001) and had substantially worse QoL (r_s_ = -.407, p < .001), as shown in **S1A–S1C Fig**.

### Comparison of 1-min STST performances across HRQoL classes

**Fig 1:**
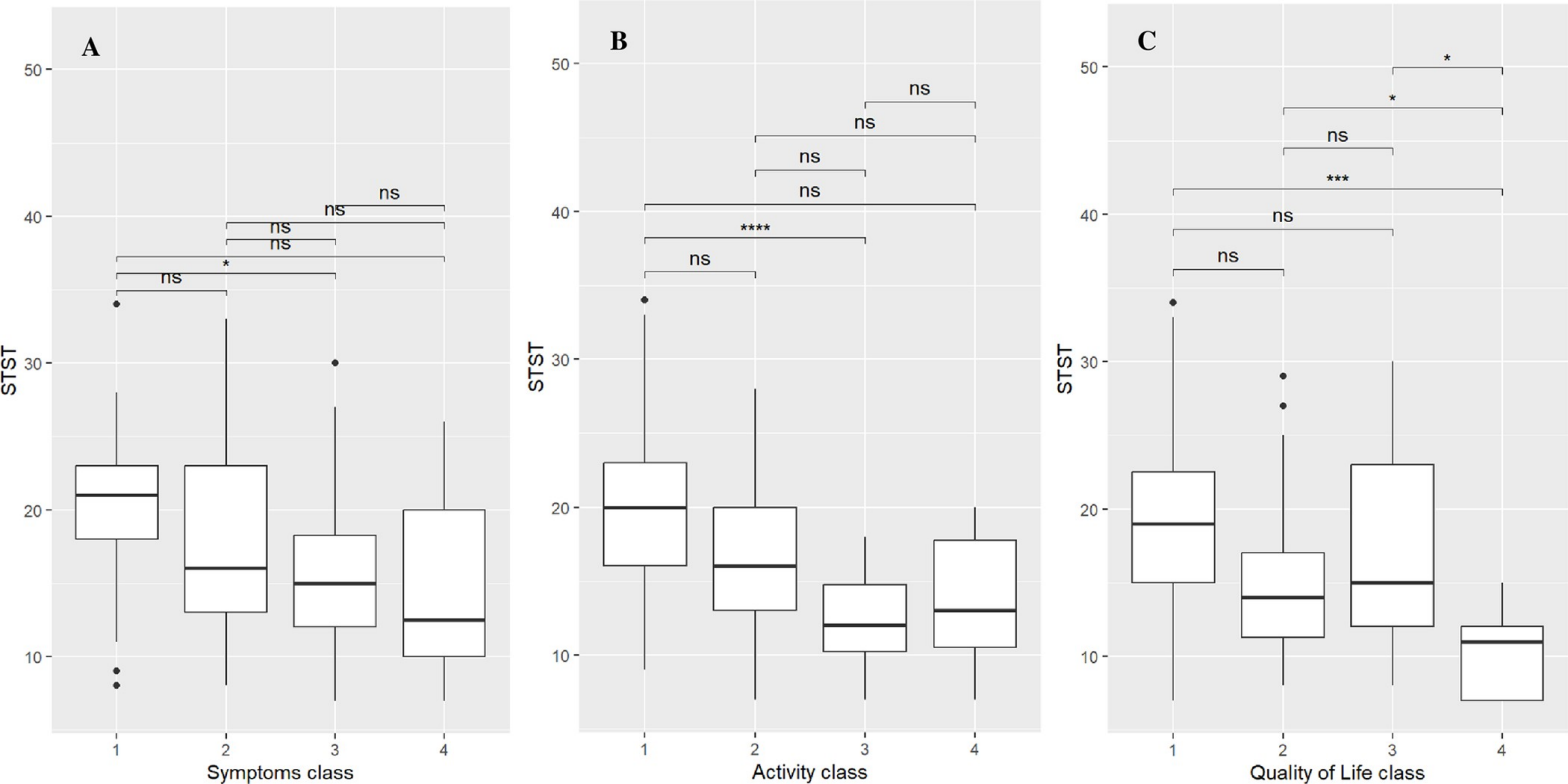
A-C. Box plots showing the distribution of 1-min STST performances across HRQoL classes. Health-related quality of life (HRQoL) scores are organized by classes depending on if a patient reported mild (class 1), moderate (class II), severe (class III) or very severe (class IV) HRQoL impairment. Each box chart displays the following information: The heavy central line is the median value; the bottom and top lines of the box are the first and third quartiles of data and individual dots are outliers.Differences in 1-min STST performance between classes are coded by significance stars (*: *p* < .05, **: *p* < .01, ***: *p* < .001). **Abbreviation.** STST: Sit-to-stand test.

As shown by box plots in **Fig 1A–1C**, the 1-min STST performances varied across classes for all subcategories. In detail, there was a significant difference in 1-min STST performance between class I and III (p = .047) (**Fig 1A**) in the symptom subcategory. There was a significant decrease in the 1-min STST performance from class I to class III (p < .001) (**Fig 1B**) in the activity limitation subcategory.

Concerning the QoL subcategory, patients in class IV had a significantly worse 1-min STST performance compared to patients in class I (p < .001), class II (p = .029) and class III (p = .021) (**Fig 1C**).

### Linear regression analysis

Results of univariate and multivariable linear regression analysis are displayed in **Table 2**. In univariate regression analysis, the 1-min STST performance was associated with CAMPHOR scores in the subcategories symptoms (est. β = -0.119, 95%, p < .001), activity limitations (est. β = -0.254, p < .001) and QoL (est. β = -0.139, p < .001).

Further, in multivariable linear regression analysis, adjusted for age, sex, BMI and mPAP, a lower 1-min STST performance was significantly related to a higher symptom burden (est. β = -0.112, p = .003), more limitations in activities of daily living (est. β = -0.198, p < .001) and a greater reduction of QoL (est. β = -0.130, p < .001) assessed with the CAMPHOR questionnaire.

**Table 2:** Univariate and multivariable linear regression analysis for the prediction of HRQoL subcategories using Rasch transformed scores.

		Univariate analysis							Multivariable analysis				
		Est. β	95% CI		p-value			Est. β		95% CI		p-value	
	Variable		lower	upper			Variable			lower	upper		
Symptoms	1-min STST	-0.119	-0.179	-0.059	**< .001**		1-min STST	-0.112		-0.185	-0.040	**.003**	
							Age	-0.010		-0.041	0.022	.551	
							Female sex	0.286		-0.531	1.103	.488	
							BMI	0.003		-0.059	0.065	.929	
							mPAP	0.027		-0.007	0.062	.121	
		R^2^ = 0.151		F = 15.338				R^2^ = 0.145			F = 3.959		
		Df 1 = 1 Df2 = 86				p < .001		Df1 = 5 Df 2 = 82					p = .003
Activities	1-min STST	-0.254	-0.335	-0.172	**< .001**		1-min STST	-0.198		-0.297	-0.099	**< .001**	
							Age	0.035		-0.009	0.079	.119	
							Female sex	0.187		-0.899	1.273	.733	
							BMI	-0.007		-0.089	0.074	.859	
							mPAP	0.038		-0.005	0.082	.083	
		R^2^ = 0.290		F = 38.469				R^2^ = 0.285			F = 8.557		
		Df1 = 1 Df2 = 94				p < .001		Df1 = 5 Df2 = 90					p < .001
QoL	1-min STST	-0.139	-0.193	-0.085	**< .001**		1-min STST	-0.130		-0.196	-0.065	**< .001**	
							Age	-0.008		-0.036	0.021	.604	
							Female sex	0.174		-0.550	0.899	.634	
							BMI	-0.011		-0.066	0.044	.691	
							mPAP	0.027		-0.002	0.056	.071	
		R^2^ = 0.219		F = 26.148				R^2^ = 0.220			F = 6.302		
		Df1 = 1 Df2 = 93				p < .001		Df1 = 5 Df2 = 89					p < .001

Univariate and multivariable regression analysis of parameters possibly associated with health-related quality of life subcategories using unstandardized coefficients (estimated β) and a 95% confidence interval (CI).

**Abbreviations.** HRQoL = health-related quality of life; Est. β = estimated beta; CI = confidence interval; 1-min STST = one-minute sit-to-stand test; BMI = body mass index; mPAP = mean pulmonary artery pressure; Df = degrees of freedom; QoL = quality of life.

## Discussion

This study is the first study that describes the assessment of functional capacity, measured by the 1-min STST, and its association with HRQoL in patients with PH evaluated with the CAMPHOR questionnaire. In our study we showed that a low 1-min STST performance is associated with a poor HRQoL. We demonstrated an association of the 1-min STST performance with the individual CAMPHOR subcategories, namely symptoms, activities, and QoL, meaning that physical or functional limitations, subjective QoL and the appearance of disease related symptoms are associated with the 1-min STST performance. These findings were independent of age, sex, BMI and mPAP.

Our results describe the dynamic between a low 1-min STST performance, the appearance of PH-related symptoms and the association with a low QoL. Patients with a low 1-min STST performance tend to have more symptoms (as assessed by energy level, breathlessness, and mood), more limited activities of daily living and a worse QoL (as indicated by the extent to which PH patients are able to satisfy their needs, including socialization, role, acceptance, self-esteem, independence and security).

Until now, data on the association of the 1-min STST and HRQoL in patients with PH are sparse. Similar to our findings, the study by Reis et al. reported a strong correlation between functional capacity, assessed with the 6MWT, and HRQoL, evaluated with the CAMPHOR questionnaire, with high correlation coefficients observed for each subcategory [13]. Furthermore, a significant association between disease severity, measured by the WHO functional class, and HRQoL was shown, noting that individuals classified as WHO functional class III-IV exhibited markedly elevated HRQoL scores [13]. This aligns with our findings, given that a substantial portion of our study participants were in WHO functional class III (50%) with the perquisite that the 6MWT can be used interchangeably with the 1-min STST. By utilizing our prospective study design, allowing for “real-time” measurements and consecutive data collection, we may identify relationships between high CAMPHOR scores, increased disease severity and the results of both functional tests in the future. This endeavor might be realizable after previously demonstrating the convergent validity of both functional tests in patients with PH [33].

Besides applications in PH, previous studies across diverse disease entities have shown an association between HRQoL and functional capacity. A strong relationship between the 1-min STST performance and HRQoL was reported in patients with chronic obstructive pulmonary disease [34]. Furthermore, the number of achieved 1-min STST repetitions was moderately associated with HRQoL in another chronic obstructive pulmonary disease collective [35]. Similarly, an association of the 1-min STST performance and HRQoL, assessed with the CAMPHOR questionnaire, was found in a study with heart failure with preserved ejection fraction patients [20].

In addition to its correlation with HRQoL, prior studies showed that the 1-min STST is also associated with other measurements used to assess patients with PH such as the WHO-FC [33, 36], NT-proBNP [33, 36], mPAP [33] and echocardiographic markers of right ventricular function [20, 37]. Since the ability to stand up from a chair reflects a gesture performed multiple times in daily living, a better performance in the 1-min STST implies less difficulty in performing daily physical activities. Furthermore, a better performance may indicate a lower impact of the disease on symptoms and QoL. These features of the 1-min STST and its strong correlation with the distance walked in the 6MWT [21, 33], suggest the potential for its use as a surrogate for the 6MWT and as a measure of HRQoL in patients with PH.

The HRQoL assessment via the CAMPHOR questionnaire is supported by current guidelines for diagnosis and treatment of PH [8]. However, up to now the CAMPHOR questionnaire has limited application in routine clinical practice given its length (65 items, estimated completion time of 10 minutes [38]), cost, and its limited availability. Adding up, on the correlation between the 1-min STST and HRQoL, it has been shown, that HRQoL is associated with survival in several studies [39–42]. Therefore, the simple and short test-design of the 1-min STST may enable prognostic statements. However, this must be evaluated in further studies.

The 1-min STST is especially useful for people with unsteady gait and balance problems, which are common in elderly patients, or for those who rely on ambulatory devices such as canes, crutches, walkers or on oxygen apparatuses. It enables a location-independent, time-efficient testing and is thus especially useful in an outpatient setting and may be used as additional screening tool in PH patients.

The findings of our study may have direct implications for clinical practice and future research. The 1-min STST can be easily performed in clinical settings in patients with PH and it may provide valuable information for clinicians in patient care. It may help to identify areas of impairment and tailor interventions to improve HRQoL. Interventions such as structured and supervised exercise training (endurance and strength training) have been shown to be beneficial for patients with PH as they improve exercise capacity, muscle function, pulmonary circulation and QoL [43, 44]. Subjective physical exertion, measured with the BDS, a peripheral oxygen saturation below 85% and other safety cut-offs (as outlined by the ERS statement [45]) provide a safe way to monitor exercise training in PH patients. The STST is known to estimate and monitor functional exercise performance in other disease entities [19, 29, 46] and therefore the use of the STST may further improve the monitoring of exercise interventions and estimate exercise capacity in PH patients.

### Future directions

Future directions could involve the exploration of alternative assessments of functional capacity beyond the 1-min STST. Assessments such as cardiopulmonary exercise testing (CPET) [47], the Timed Up and Go test [48] and the stair-climbing test [49] have the potential to provide additional insights into the relationship between HRQoL and functional capacity. However, it is important to consider the potential challenges associated with alternative tools. Tools like CPET may not be as cost-effective as the 1-min STST. Furthermore, the feasibility and practicality of these assessments for patients with PH need careful consideration, as some tests may be more challenging to perform. Future research could focus on addressing these limitations, guiding the development of cost-effective alternatives. This approach may contribute to a more comprehensive understanding of patients’ functional capacity in relation to HRQoL facilitating tailored assessments in clinical settings, ultimately leading to improvements in patient management for patients with PH.

### Study limitations

This study had some limitations. First, the study was conducted at a single center and the sample size was relatively small. Therefore, the findings may not be applicable to the general PH population. Second, this study was cross-sectional in design and therefore, causal inferences cannot be made. Further studies are needed to investigate the longitudinal association between the 1-min STST and HRQoL in patients with PH. Third, the study did not examine other potential factors that may influence the relationship between the 1-min STST and HRQoL, such as comorbidities and motivational aspects. These other factors warrant further exploration. In addition, despite the careful selection, there was no control group. Multicenter studies with a bigger sample size including a control sample should complement the data obtained.

## Conclusion

Our study showed that the 1-min STST performance was associated with all CAMPHOR HRQoL subcategories in patients with PH. We suggest incorporating the 1-min STST into the routine evaluation of patients with PH to assess their functional capacity correlated to their HRQoL.

## Supporting information

S1 Fig **A-C.** Association of 1-min STST results with CAMPHOR subcategory scores. Scatter plot of the association of 1-min STST results with CAMPHOR subcategory scores. **Abbreviations.** 1-min STST = one-minute sit-to-stand test; QoL = quality of life.
(DOCX)

## Abbreviation

1-min STST: One-Minute Sit-To-Stand Test
6MWT: Six-Minute Walking Test
BDS: Borg Dyspnea Scale
BMI: Body Mass Index
CAMPHOR: Cambridge Pulmonary Hypertension Outcome Review
CPET: Cardiopulmonary Exercise Testing
ESC: European Society of Cardiology
ERS: European Respiratory Society
HRQoL: Health Related Quality of Life
mPAP: Mean Pulmonary Artery Pressure
NT-proBNP: N-Terminal Prohormone of Brain Natriuretic Peptide
QoL: Quality of Life
WHO-FC: World Health Organization Functional Class
