# Supplementary material for: The one-minute sit-to-stand-test performance is associated with health-related quality of life in patients with pulmonary hypertension
Source: PLoS One. 2024 May 29;19(5):e0301483. doi: 10.1371/journal.pone.0301483 (PMC11135673; doi:10.1371/journal.pone.0301483)
Supplement: S1 Fig — A-C. Association of 1-min STST results with CAMPHOR subcategory scores. Scatter plot of the association of 1-min STST results with CAMPHOR subcategory scores. Abbreviations. 1-min STST = one-minute sit-to-stand test; QoL = quality of life. (DOCX) [file pone.0301483.s001.docx]

**S1A-C Fig.** Association of 1-min STST results with CAMPHOR subcategory scores

**B**

**A**


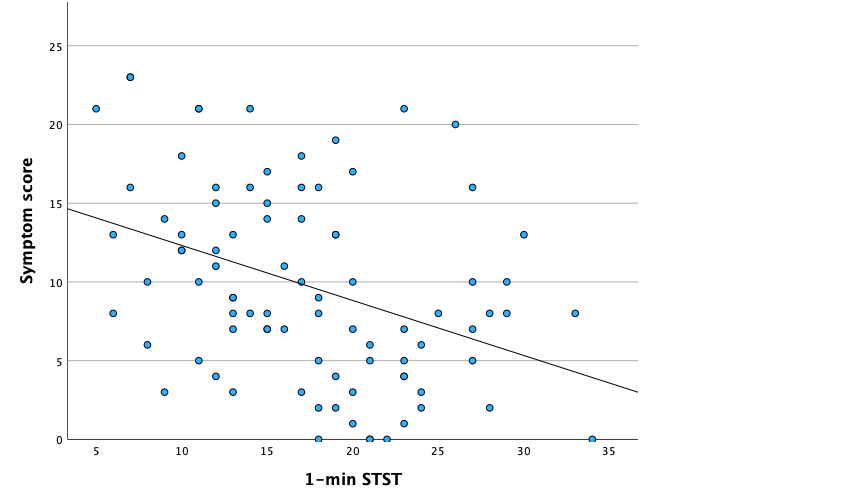

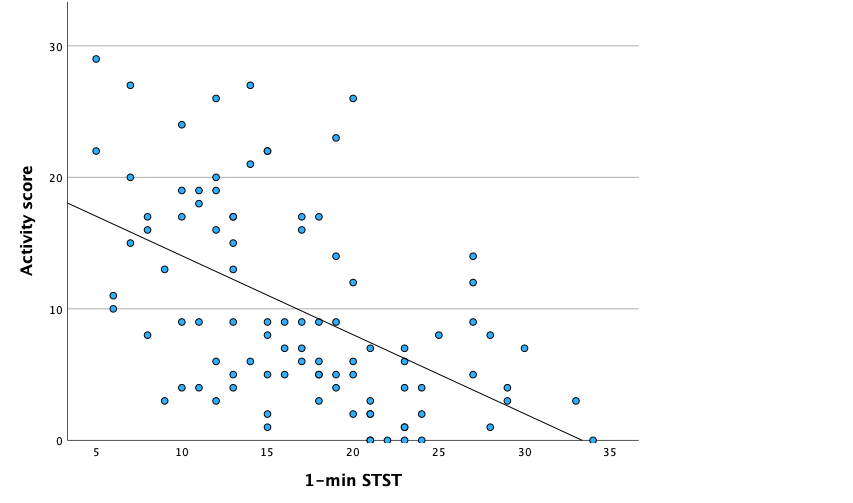


**r_s_ = -.551**

**p <0.001**

**r_s_ = -.398**

**p <0.001**

**C**


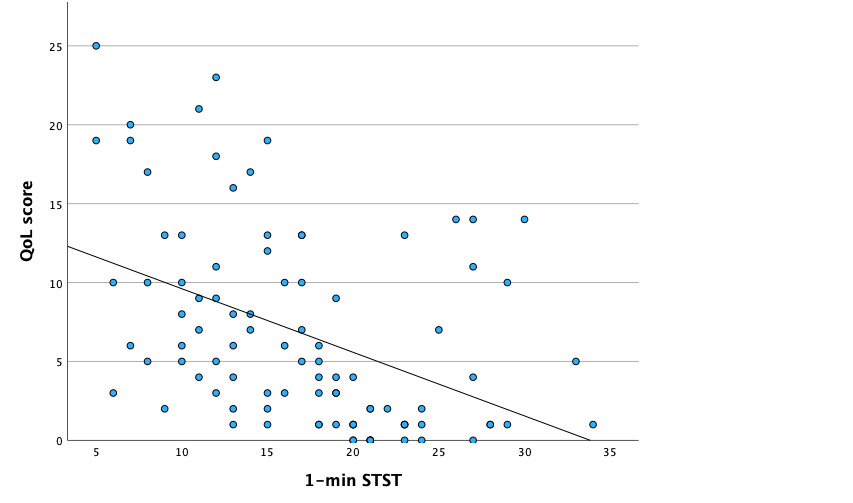


**r_s_ = -.407**

**p <0.001**

Scatter plot of the association of 1-min STST results with CAMPHOR subcategory scores

**Abbreviations.** 1-min STST = one-minute sit-to-stand test; QoL = quality of life.
